# Can network biology unravel the aetiology of congenital hyperinsulinism?

**DOI:** 10.1186/1750-1172-8-21

**Published:** 2013-02-08

**Authors:** Adam Stevens, Karen E Cosgrove, Raja Padidela, Mars S Skae, Peter E Clayton, Indraneel Banerjee, Mark J Dunne

**Affiliations:** 1 Department of Endocrinology, Royal Manchester Children's Hospital, Oxford Road, Manchester, M13 9WL, UK; 2 Faculty of Life Sciences, University of Manchester, Manchester, M13 9PT, UK

## Abstract

Congenital Hyperinsulinism is a condition with a number of genetic causes, but for the majority of patients, the underlying aetiology is unknown. We present here a rational argument for the use of computational biology as a valuable resource for identifying new candidate genes which may cause disease and for understanding the complex mechanisms which define the pathophysiology of this rare disease.

## Letter

Congenital Hyperinsulinism (CHI) is a rare disease, but is the most common cause of recurrent hypoglycaemia in infancy [1]. The treatment of CHI can be difficult and involves drugs which may not be successful and often are poorly tolerated. As a potentially life-threatening condition, CHI is associated with lifelong sequelae - including critical brain damage (epilepsy, cerebral palsy and neurological impairment) in up to 40% of cases. To date, nine candidate genes associate with CHI, but for the majority of patients – estimated to be approximately 65%, both the aetiology of the CHI and the mechanisms of disease are unknown.

Our current approach to the classification and treatment of CHI is based largely upon observational correlations between the pathological analysis of candidate gene defects and clinical symptoms of hypoglycaemia [1-3]. In this respect, there are similarities between CHI and many other diseases in which numerous mutations in different genes give rise to clinical phenotypes that are essentially indistinguishable from one another. However, under normal physiological conditions, cells function correctly because there is a high degree of interdependency between individual biochemical components (DNA, RNA, proteins and metabolites) and their complex interactions (DNA-protein interactions, protein-protein interactions, metabolic and biochemical pathways, etc.), and tissues function in a co-ordinated manner because there is interplay between different cell types. Diseases rarely result from an abnormality in a single gene, but are in fact the manifestation of disturbances in the multiple networks that integrate cellular processes, and those that link cells with tissues, and tissues with organ systems. As a result, current approaches to molecular diagnosis, however valuable, have shortcomings. These include a lack of sensitivity in identifying preclinical disease, a poor ability to predict prognosis, and ambiguity in defining and resolving a condition where several clinical phenotypes can be observed. All of these inadequacies are evident in CHI, with our current understanding of the causes of disease failing to distinguish transient from persistent disease at the point of presentation and to determine accurately the severity of disease. Also, it is not possible to identify at diagnosis which patients require curative surgery from those who could be successfully managed by short or long-term medical therapy. For these reasons we believe an innovative approach to CHI is required – one which can identify new causes and new mechanisms of dysfunction. One such approach is the use of network biology, first to summate the various interactions and interdependencies between gene networks, and second to identify critical components and pathways which may contribute to the pathophysiology of CHI.

**Figure 1:**
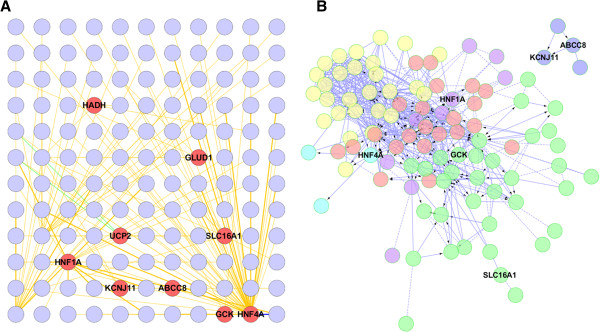
**Human interactome network analysis of CHI-associated genes.** (**A**) Genes with a known association with CHI in red were used to infer a network from the BioGRID model of the human interactome (http://thebiogrid.org/). (**B**) Biological pathway ontology associated with each network module has been colour coded for clarity and listed separately in Table 1; hypergeometric test false discovery rate (FDR) ≤ 0.001. The inferred network was generated using the BioGRID plugin (version 3.1.94) for Cytoscape (version 2.8.3) to identify the primary interactors of CHI genes from all known physical and genetic evidence. The CHI gene associated network inferred from the BioGRID interactome model was then imported into the Reactome Plugin for Cytoscape and modularity was analysed using spectral partition clustering [11] .

Today, a number of diseases are being redefined through the combination of contemporary molecular techniques (e.g. expression arrays, epi/genomic analysis, proteomic and metabolomics analysis) and bioinformatics to describe the interconnected networks that govern normal cellular function and are involved in the pathophysiology of disease [4-6]. First published in 2007, the “*human disease network*” describes a concept in which a specific genetic abnormality not only alters the product that it encodes, but also changes the activity of the network to which it belongs [7]. This change in activity can spread along intra- and intercellular links of a network of interconnectivity and in doing so it will also alter the activity of genes or other biochemical products that otherwise carry no abnormalities [7,8]. A consequence of this widely-held hypothesis is that the interdependencies among molecular components will form strong functional and causal relationships which can be used to map diseases in the form of “disease networks”. With an estimated excess of 100,000 “nodes” representing genes, RNA, proteins, and metabolites, and an even higher number of “links” representing the functionally-relevant interactions between the nodes (including DNA-protein interactions, DNA-chemical interactions, protein-protein interactions, metabolic / biochemical pathways, etc.), the scale and complexity of this network – the “*human interactome*”, is daunting. However, many emerging studies now describe how signature changes in the expression or activities of nodes as a consequence of disease can be integrated within the human interactome and that this relationship can then be used to unlock some of the complexities of pathophysiology [4-8]. With this in mind, we have recently analysed the bioinformatics of the nine known CHI-causing genes – which range from transcription factors to metabolic enzymes and solute transporters. This was performed by generating an interaction network seeded by the known CHI-associated genes (*GLUD, SLC16A1, HADH, UCP2, KCNJ11, ABCC8, HNF1A, GCK, HNF4A*) and their physical and genetic interactors as identified using the BioGRID interactome model [9]. Surprisingly, we found that this diverse range of genes will form a core network – described here as the CHI-Disease Network, composed of highly connected members, Figure 1. There is increasing evidence that the functions associated with a biological network correlate with modular structure within the network [10]. We therefore assessed the presence of modularity in the derived CHI related network using spectral-partition clustering [11] and assessed known biological pathways present in these modules, Table 1.

**Table 1:** Pathway ontology associated with the CHI disease network

**Network module**	**Biological pathway**	**FDR**
Cellular Signalling	Tropomyosin Receptor Kinase Signalling	5.0 × 10^-5^
	RAF/MAP Kinase Cascade	5.3 × 10^-5^
	Neurotrophin Signalling	5.9 × 10^-5^
	mTOR Signalling	6.3 × 10^-5^
	Syndecan-1-mediated Signalling	4.0 × 10^-4^
	TRAIL Signalling	3.0 × 10^-4^
Nuclear Signalling	Regulation of SMAD2/3 Signalling	1.0 × 10^-4^
	Oestrogen Receptor-α Signalling	1.1 × 10^-4^
	Oestrogen Receptor-β Signalling	1.5 × 10^-4^
	Retinoic Acid Receptor Signalling	1.4 × 10^-4^
Growth Factor Signalling	BARD1 Signalling Events	1.3 × 10^-4^
	p53 Signalling pathway	2.0 × 10^-4^
	HDAC Class III Signalling	1.0 × 10^-3^
	TGFβ Signalling	2.6 × 10^-4^
Development	ErbB2/ErbB3 Signalling	6.6 × 10^-3^
	Presenilin Signalling	9.0 × 10^-3^
	GMCSF-Mediated Signalling	6.6 × 10^-3^
Function	Integration of Energy Metabolism	1.0 × 10^-3^

Five network modules associate with the CHI Disease Network (Figure 1) and each module has nodes which are represented in a number of canonical biological pathways (http://www.ncbi.nlm.nih.gov/biosystems/). Several of these have been highlighted for each module along with their False Discovery Rate (FDR) which represents the chance occurrence of nodes being co-located with pathways.

This is the first analysis of its kind for CHI or any other monogenic disorder of glucose-regulation in infancy or childhood (e.g. neonatal diabetes mellitus, MODY, etc.). From here, we are now in a position to explore the possibility that genes integral to the network or tightly connected to it, may be new candidates for the aetiology of CHI and other monogenic causes of glucose-regulation disorders. Furthermore, by analysis of the components of the associated pathway ontology – listed in Table 1, we have created a portal to identify novel mechanisms of disease which may provide insights for the management and treatment of CHI. We strongly believe that these *in silico* techniques involving access to readily available databases are highly applicable to many rare diseases, and that when used alone or in combination with other datasets – e.g. metabolomic, they will form the future basis of identifying of new candidate gene defects, and understanding the pathophysiology of rare diseases.
